# Murine Gammaherpesvirus-68 Inhibits Antigen Presentation by Dendritic Cells

**DOI:** 10.1371/journal.pone.0001048

**Published:** 2007-10-17

**Authors:** Christopher M. Smith, Michael B. Gill, Janet S. May, Philip G. Stevenson

**Affiliations:** Division of Virology, Department of Pathology, University of Cambridge, Cambridge, United Kingdom; Institut Pasteur, France

## Abstract

Dendritic cells (DCs) play a central role in initiating adaptive immunity. Murine gammaherpesvirus-68 (MHV-68), like many persistent viruses, infects DCs during normal host colonization. It therefore provides a means to understanding what host and viral genes contribute to this aspect of pathogenesis. The infected DC phenotype is likely to depend on whether viral gene expression is lytic or latent and whether antigen presentation is maintained. For MHV-68, neither parameter has been well defined. Here we show that MHV-68 infects immature but not mature bone marrow-derived DCs. Infection was predominantly latent and these DCs showed no obvious defect in antigen presentation. Lytically infected DCs were very different. These down-regulated CD86 and MHC class I expression and presented a viral epitope poorly to CD8^+^ T cells. Antigen presentation improved markedly when the MHV-68 K3 gene was disrupted, indicating that K3 fulfils an important function in infected DCs. MHV-68 infects only a small fraction of the DCs present in lymphoid tissue, so K3 expression is unlikely to compromise significantly global CD8^+^ T cell priming. Instead it probably helps to maintain lytic gene expression in DCs once CD8^+^ T cell priming has occurred.

## Introduction

Persistent viruses commonly infect dendritic cells (DCs); epidemic viruses seem to do so more rarely. The pivotal role DCs play in initiating anti-viral immunity means that virus-infected DCs potentially elicit a rapid and potent immune attack [1], [2]. Thus, why persistent viruses should infect them is not clear. The chance to exploit DC functions presumably offers a selective advantage that outweighs the risk of greater immunogenicity. A key factor in the cost/benefit balance of DC infection is MHC class I-restricted antigen presentation. Viral evasion proteins are therefore likely to be important in infected DCs both to promote DC survival and to off-set the increased opportunities for immune priming.

Gamma-herpesviruses infect lymphocytes and therefore have a particularly intimate relationship with host immune function. Murine gamma-herpesvirus-68 (MHV-68) is a natural murid parasite [3] related to the Kaposi's Sarcoma-associated Herpesvirus (KSHV) [4]. Like KSHV [5], MHV-68 infects epithelial cells, B cells, macrophages and DCs [6]. Both viruses inhibit MHC class I-restricted antigen presentation [7]. The MHV-68 K3 ubiquitinates MHC class I heavy chains [8], [9] and TAP [10]. The KSHV K3 and K5 function similarly [11], [12] to down-regulate a range of immune signalling molecules, including MHC class I [7], [13], [14], ICAM-1 and CD86 [15]. Gamma-herpesviruses match their CD8^+^ T cell evasion to specific gene expression programs. Thus MHV-68, like Epstein-Barr virus [16], [17], evades antigen presentation in cis during episome maintenance [18] when K3 is probably not transcribed [19]. Proliferating, latently infected germinal centre B cells do transcribe K3, and K3 disruption causes a CD8-dependent defect in viral latency amplification [20]. Latency-associated K3 expression has now also been identified for KSHV [21]. Transactivation of the MHV-68 K3 promoter by the ORF50 lytic switch protein [22] suggests that K3 has an another important and as yet undefined function in lytic infection. MHV-68-infected DCs transcribe K3 and appear to support a mixture of lytic and latent infection [23]. Thus, K3 is made either in latently infected DCs or when their virus reactivates. MHV-68 infects DCs when primed, virus-specific CD8^+^ T cells are abundant [24]. Such CD8^+^ T cells normally eliminate DCs that present viral antigens [25]. K3 may therefore be important for the survival of lytically infected DCs. Because MHV-68-infected mice contain very few recoverable infected DCs [23], in vitro analysis is necessary to define cell phenotypes. A key task is to distinguish lytic from latent infection. Viral gene expression differs radically between these states, so it should not be surprising if DC phenotypes did too.

Distinguishing lytic and latent infections in mixed cultures depends critically on suitable assays. Lytic and latent infections of ex vivo cells are typically distinguished by disrupting cells or not to stop reactivation, and then co-culturing them with permissive fibroblasts [23], [26]. Such assays depend on infectious virions being sparse, and become problematic when large amounts of pre-formed infectious virus have been added to in vitro cultures. PCR-based quantitation is similarly prone to be confounded by input virus and infected cell debris. A second problem is that such assays detect only population averages. Thus, they cannot distinguish which cells are responsible for what effects in mixed populations. Non-responsive cells may consequently be missed because those making measurable responses dominate the readout. For example, a few latently infected or uninfected DCs may secrete a lot of cytokines while lytically infected DCs secrete none.

Flano et al. [27] have concluded that K3 does not function in MHV-68-infected DCs. However, they did not distinguish lytic from latent infection, made no comparison of K3^-^ and K3^+^ viruses, and did not establish whether the virus they used even retained K3-such immune evasion functions are frequently lost with in vitro passage. We have examined DC antigen presentation by distinguishing lytic from latent infections and comparing K3^−^ and K3^+^ viruses. We find that MHV-68 markedly down-regulates antigen presentation in lytically infected DCs and that this is largely if not entirely dependent on K3. MHV-68 also down-regulated CD86 expression by a K3-independent mechanism. Lytically and latently infected DCs were phenotypically very different. Mature DCs appeared to resist infection altogether. Besides demonstrating that CD8^+^ T cell evasion has an important function in MHV-68-infected DCs, our data highlight the problems associated with drawing conclusions from mixed infected cultures, and suggest that some of the phenotypes reported for MHV-68-exposed DCs need to be re-examined.

## Materials and Methods

### Cells

BHK-21 cells, NIH-3T3-CRE cells [20] and the 49100.2 T cell hybridoma [28], which recognizes an H2-D^b^-restricted immunodominant MHV-68 peptide derived from ORF6 [24] were propagated in DMEM with 100 U/ml penicillin, 100 µg/ml streptomycin, 2 mM glutamine and 10% fetal calf serum. Dendritic cells were grown from bone marrow progenitors of C57BL/6 or BALB/c mice in RPMI with 10% fetal calf serum, 50 µM 2-mercaptoethanol, 100 U/ml penicillin, 100 µg/ml streptomycin and 7.5 ng/ml GM-CSF (PeproTech, Rocky Hill, NJ). Both gave similar results. Bone marrow cells were first plated onto tissue culture plastic (30 min, 37°C) and the adherent (macrophage-rich) cells discarded. The remaining cells were cultured, changing the medium every 2 d. After 3 d, non-adherent (granulocyte-rich) cells were discarded. After 7 d, the non-adherent cells (90% CD11c^+^IA^+^Gr1^−^) were harvested. Consistent levels of maturity and responsiveness to maturation signals were confirmed for each DC population by flow cytometric staining for CD11c, CD86 and MHC class II with or without prior LPS treatment. We also established that the cells were negative for the granulocyte marker GR1. CD4^+^ T cells specific for IA^d^ plus ovalbumin residues 323–339 were harvested from lymph nodes of DO.11.10 transgenic mice [29]. CD8^+^ T cells specific for H2-K^b^ plus ovalbumin residues 257–264 were harvested from lymph nodes of OT-I transgenic mice [30], kindly provided by Prof. D. Fearon.

### Viruses

All viruses were derived from a cloned MHV-68 BAC [31]. The gM-eGFP mutant has been described [32]. The MHV-68 K3 gene was disrupted on this background by digesting a SacI genomic clone (genomic co-ordinates 21383–28336) [33] with NruI (24851) and BsmI (24999). The BsmI-cut 5′ overhang was filled in with Klenow fragment DNA polymerase (New England Biolabs, Hitchin, U.K.) and the 2 blunt ends ligated together. The mutant SacI clone was then subcloned into the SacI site of pST76K-SR and recombined into the gM-eGFP^+^ MHV-68 BAC by standard methods [31]. The eGFP coding sequence was fused to the 5′ end of ORF73 by first PCR amplifying ORF73 with 5′ EcoRI and 3′ XhoI restriction sites in the respective primers and cloning the PCR product into the EcoRI/SalI sites of pEGFP-C2 (Clontech). A genomic segment (co-ordinates 104869–106108) corresponding to the region upstream of ORF73 and incorporating its splice acceptor site [34] was then amplified by PCR using NheI-restricted primers and cloned into the NheI sites of the same vector. The eGFP coding sequence with its 2 genomic flanks was then subcloned as a blunted fragment into the SmaI site of pST76K-SR and recombined into the MHV-68 BAC as before. Infectious viruses were reconstituted by transfecting BAC DNA into BHK-21 cells with Fugene-6 (Roche Diagnostics Ltd., Lewes, U.K.). Except when eGFP expression from the BAC cassette was used as a marker of infection (BAC-eGFP), the loxP-flanked cassette was removed by virus passage in NIH-3T3-CRE cells [20]. All viruses were grown in BHK-21 cells. Infected cultures were cleared of infected cell debris by low-speed centrifugation (1000×g, 3 min). Virions were then concentrated from supernatants by high speed centrifugation (38000×g, 90 min). Virus titers were determined by plaque assay on BHK-21 cells [35].

### Southern Blotting

Viral DNA was isolated from infected BHK-21 cells by alkaline lysis, phenol/chloroform extraction and salt/ethanol precipitation [35], digested with BamHI or ApaI, electrophoresed on a 0.8% agarose gel and transferred to positively charged nylon membranes (Roche Diagnostics). A ^32^P-dCTP-labelled probe (APBiotech, Little Chalfont, U.K.) was generated from either HinDIII (21965–26711) or BamHI (49938–59884) genomic fragments [36] by random primer extension (Nonaprimer kit, Qbiogene, Bingham, U.K.). Membranes were hybridised with probe (65°C, 18 h), washed to a stringency of 0.2% SSC, 0.1% SDS and exposed to X-ray film.

### Antigen presentation assays

To assay MHC class II-restricted presentation of soluble ovalbumin, DCs (2.5×10^5^/well) were infected or not (3PFU/cell, 24 h) with MHV-68, then pulsed for 2 h with ovalbumin (Sigma Chemical Co., Poole, U.K.). The cells were then washed and DO.11.10 lymph node cells added (10^6^/well). Supernatants were harvested 15 h later and assayed for IL-2 by ELISA (BD-Pharmingen, Kidlington, U.K.). To assay MHC class I-restricted presentation of soluble ovalbumin, DCs (2.5×10^5^/well) were infected or not (3PFU/cell, 24 h) with MHV-68, then pulsed for 6 h with ovalbumin. The cells were washed and CFSE-labelled (5 µM, 15 min) OT-I lymph node cells added (10^6^/well). The CD8^+^ T cells were harvested 72 h later and assayed for CFSE content by flow cytometry. To assay MHC class I-restricted presentation of the H2-D^b^-restricted MHV-68 p56 epitope [24], DCs (2.5×10^5^/well) were infected or not with MHV-68 (3PFU/cell, 4 h) and pulsed or not with 20 nM p56 peptide, washed and then incubated (18 h) with p56-specific 49100.2 T cells. The cells were then washed in PBS and lysed in PBS/5 mM MgCl_2_/1% NP-40/0.15 µM chlorophenol-red-beta-D-galactoside (Merck Biosciences, Nottingham, U.K.) to assay beta-galactosidase activity. After 2–4 h the absorbance at 595 nm was read on a Biorad Benchmark Microplate Reader.

### Immunfluorescence

Non-adherent DCs were plated onto poly-D-lysine coated coverslips after 7 days of culture, infected or not with MHV-68, then washed with PBS and fixed with 4% PFA for 10 min. The cells were then permeabilized with 0.1% Triton-X-100 for 10 min, blocked for 1 h with 3% BSA and stained for 2 h with MHV-68-specific monoclonal antibodies plus Alexa Fluor 568-conjugated anti-mouse IgG pAb (Invitrogen), or with the MHC class II-specific mAb M5/114 plus Alexa Fluor 568 anti-rat IgG pAb (Invitrogen). EGFP fluorescence was visualized directly. The MHV-68-specific mAbs used were MG-12B8 (anti-ORF65 capsid component) [32], 3F7 (anti-gN) [37], CS1-4A5 (anti-thymidine kinase) and BN-3H8 (anti-ORF75a). The cells were mounted in Prolong Gold antifade reagent with DAPI (Invitrogen) and images were taken on a Leica Confocal microscope at 63× magnification.

### Flow cytometry

DCs were detached from tissue culture plates by pipetting, washed in PBS, blocked with 3% BSA plus an anti-CD16/32 mAb, then stained for 30 min for CD11c (APC-conjugated mAb N418), plus either IA^b^ (PE-conjugated mAb AF6-120.1), CD80 (PE-conjugated mAb 16-10A1) or CD86 (PE-conjugated mAb GL1) (all from BD-Biosciences). H2-D^b^ was detected with mAb 28.14.8 plus PE-conjugated goat anti-mouse IgG pAb. The cells were analyzed on a FACS Calibur using Cellquest (BD-Biosciences). Dead cells were excluded by propidium iodide staining (1 µg/mL).

## Results

### Identification of dendritic cell infection by viral eGFP expression

**Figure 1:**
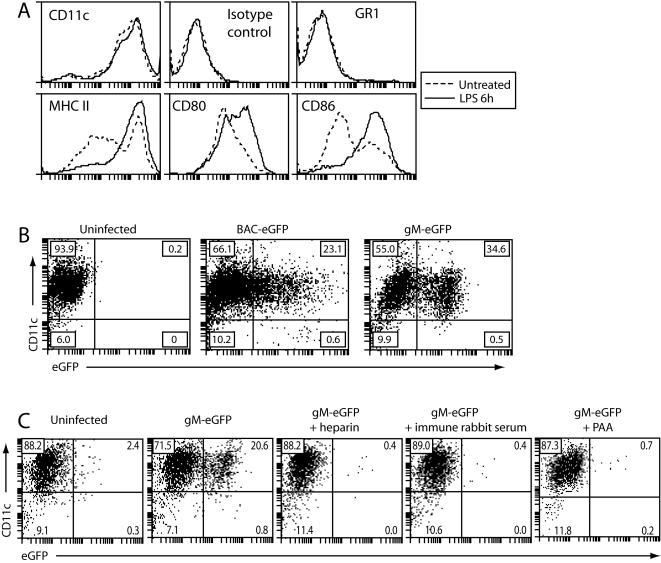
Direct identification of lytically infected DCs. A. Cells grown from C57BL/6 mouse bone marrow with GM-CSF were tested for cell surface expression of dendritic cell markers by flow cytometry, with or without 6 h LPS treatment (250 ng/mL). The data are from 1 of 5 equivalent experiments. B. Equivalent cells to A were left uninfected or infected with MHV-68 (3PFU/cell, 22 h) expressing eGFP either from a human cytomegalovirus IE1 promoter (BAC-eGFP) or fused to the endogenous gM C-terminus (gM-eGFP). Infection was evaluated by flow cytometric assay of eGFP expression. The percentage of cells in each quadrant is shown. The data are from 1 of 5 equivalent experiments. C. Equivalent cells to A were left uninfected or infected with gM-eGFP MHV-68 (3PFU/cell, 22 h). Infections were done in the presence of 100 µg/ml heparin, 0.1% MHV-68-immune rabbit serum or 100 µg/ml phosphonoacetic acid (PAA) as shown. Infection was assessed by flow cytometric assay of gM-eGFP expression.

DCs were grown from bone marrow precursors by standard methods (Fig. 1A). BAC-derived MHV-68 retaining its loxP-flanked BAC cassette [31] includes eGFP under a human cytomegalovirus (HCMV) IE-1 promoter (BAC-eGFP); such HCMV IE-1 promoter-driven gene expression is often used to track herpesvirus infections. We compared this infection marker with MHV-68 carrying an eGFP tag on the endogenous gM C-terminus (gM-eGFP) [32], [38] (Fig. 1B). Using an infection multiplicity of 3PFU/cell, approximately 20% of CD11c^+^ DCs were BAC-eGFP^+^ after 18 h and approximately 30% of CD11c^+^ cells were gM-eGFP^+^.

MHV-68 infection of fibroblasts is highly sensitive to inhibition by soluble heparin [35], [39]. Heparin also blocked gM-eGFP expression in DCs (Fig. 1C), as did an MHV-68-immune rabbit serum [26] or phosphonoacetic acid, which inhibits viral late gene expression (Fig. 1C). DC infection therefore proceded by a glycosaminoglycan-dependent pathway much like that described for fibroblasts and epithelial cells [35], [39].

### gM-eGFP expression but not HCMV IE1 promoter-driven eGFP expression marks DCs as lytically infected

**Figure 2:**
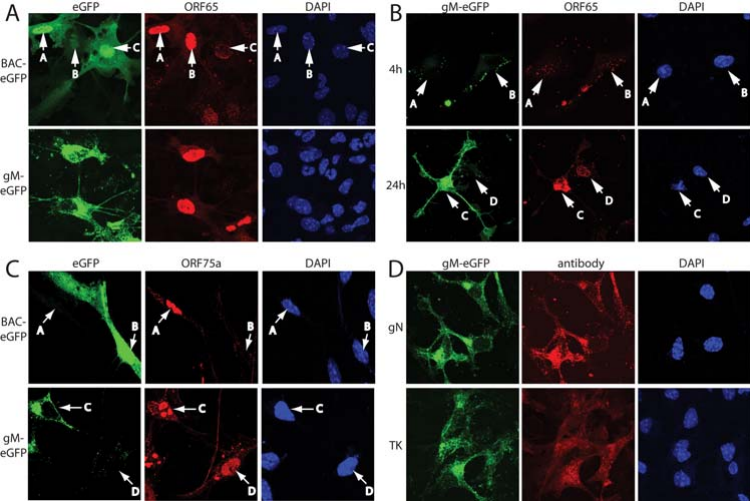
Correlating BAC-eGFP and gM-eGFP expression with other markers of MHV-68 lytic infection in DCs. A. DCs were plated onto coverslips and infected (3PFU/cell, 22 h) with BAC-eGFP or gM-eGFP MHV-68 as shown, then fixed, permeabilized and stained for the ORF65 capsid component with mAb MG-12B8. Nuclei were visualized with DAPI. The data are from 1 of 3 equivalent experiments. B. DCs were infected (3PFU/cell) with gM-eGFP MHV-68 and then washed and fixed after 4 h or 24 h before staining for ORF65 as in A. The data are from 1 of 3 equivalent experiments. C. DCs were infected as in A and stained for ORF75a with mAb BN-3H8 The data are from 2 of 3 equivalent experiments. D. DCs were infected with gM-eGFP MHV-68 as in A and stained for gN with mAb 3F7 or for thymidine kinase with mAb CS-4A5. The data are from 1 of 2 equivalent experiments.

We wanted first to establish whether a given DC was lytically or latently infected. We therefore correlated virus-driven eGFP expression with capsid distribution. Incoming MHV-68 capsids migrate to the nuclear margin but remain perinuclear, whereas newly expressed capsids assemble inside the nucleus [32]. As with other herpesviruses [40], secondary envelopment and mature virion egress are rapid, so new MHV-68 capsids in the cytoplasm are rare [35]. Thus, punctate, perinuclear ORF65 capsid staining reflects input virions, which may establish either lytic or latent infection, while strong intranuclear ORF65 staining reflects lytic infection [32], [38]. BAC-eGFP expression in DCs did not correlate with nuclear ORF65 staining (Fig. 2A). Arrow A shows strong eGFP expression in a lytically infected DC, arrow B shows very weak eGFP expression in a lytically infected DC, and arrow C shows a DC with strong eGFP expression but only input capsids. BAC-eGFP^−^ cells with perinuclear capsids were also evident and each staining pattern was common (>10% of all MHV-68-exposed DCs). These data were consistent with MHV-68 infected macrophages being either BAC-eGFP^+^ or BAC-eGFP^−^ when supporting either lytic or latent viral gene expression [38].

In contrast, gM-eGFP^+^ cells invariably showed nuclear capsid staining (Fig. 2A). At 4h post-infection, almost all DCs contained perinuclear capsids. They had evidently also endocytosed eGFP-labelled gM (Fig. 2B, arrows A and B). The different distributions of capsid and gM-eGFP were consistent with a post-fusion migration of capsids towards nuclear pores [32]. No cells showed nuclear capsid staining at 4 h post-infection. After 24 h, nuclear capsid staining was evident in cells that showed strong gM-eGFP expression (arrow C). Capsid staining in gM-eGFP^−^ cells remained perinuclear, consistent with latent infection (arrow D). Since gM is a late gene product, it might be argued that BAC-eGFP expression could reflect early lytic infection. However, it showed no obvious correlation - either positive or negative - with staining for ORF75a (Fig. 2C), an early gene product [41]. Some DCs expressed both BAC-eGFP and ORF75a, but as illustrated in Fig. 2C, BAC-eGFP^-^ cells with nuclear ORF75a staining (arrow A) and BAC-eGFP^+^ cell without ORF75a staining (arrow B) were both abundant. In contrast, all gM-eGFP^+^ cells were also ORF75a^+ ^(Fig. 2C, arrow C). Approximately 10% of ORF75a^+^ cells expressed little gM-eGFP (Fig. 2C, arrow D). These were presumably in early lytic infection. In addition to ORF65 and ORF75a, gM-eGFP expression correlated well with gN and thymidine kinase expression in infected DCs (Fig. 2D), while BAC-eGFP expression correlated with neither (data not shown).

In summary, DCs infected with BAC-eGFP MHV-68 could be eGFP^+^ or eGFP^−^ when expressing lytic gene products and eGFP^+^ or eGFP^−^ when not expressing lytic gene products. The HCMV IE1 promoter was therefore regulated independently of the rest of the MHV-68 genome. At least as many cells must be infected as are BAC-eGFP^+^, but BAC-eGFP expression did not identify all infected cells and emphatically did not distinguish lytic from latent infection. This limitation needs to be borne in mind for the HCMV IE1-driven transcription of any antigen or marker protein from the MHV-68 genome. In contrast, gM-eGFP expression allowed us specifically to identify lytically infected DCs.

### MHV-68 is mainly latent in bone marrow-derived DCs

MHV-68 virions with eGFP-tagged gM are sufficiently fluorescent to be detected on or in infected cells even without new viral gene expression [32]. In Fig. 1B, at least 90% of DCs had endocytosed enough gM-eGFP^+^ virions for low-level fluorescence (compare with uninfected cells), but only 30% supported new lytic gene expression. The fraction of cells showing lytic gene expression increased with time and most of the DCs in infected cultures died within 2 weeks. This argued against abortive infection, and suggested that the majority of MHV-68-exposed DCs become latently infected but that their virus then quite rapidly reactivates.

**Figure 3:**
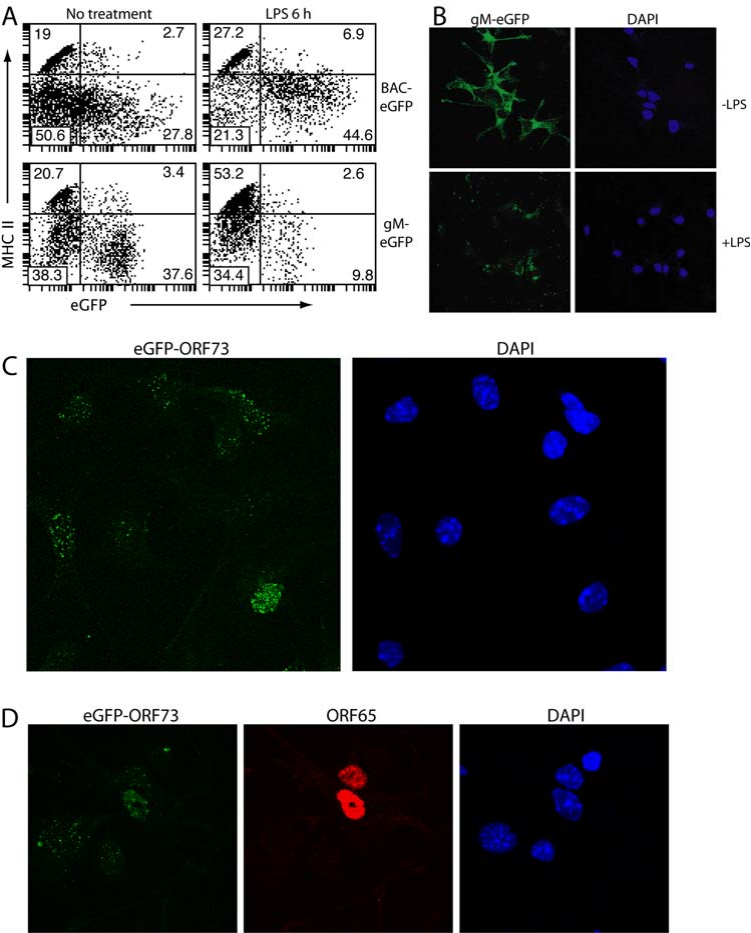
Identification of latent MHV-68 in DCs. A. DCs were infected with BAC-eGFP or gM-eGFP MHV-68 (3PFU/cell, 18 h), then treated or not with LPS (250 ng/mL) for 6 h. CD11c^+^ cells were then analyzed for eGFP expression and cell surface MHC class II expression by flow cytometry. The data are from 1 of 3 equivalent experiments. B. DCs were plated onto coverslips then infected with gM-eGFP MHV-68 and exposed or not to LPS as in A. EGFP expression was visualized directly and nuclei counterstained with DAPI. The data are from 1 of 3 equivalent experiments. C. DCs were infected (3PFU/cell, 22 h) with MHV-68 expressing eGFP-tagged ORF73, then examined by confocal microscopy. Essentially every adherent cell expressed some nuclear eGFP, although the precise staining pattern differed between individual cells. The data are from 1 of 3 equivalent experiments. D. Cells were infected with eGFP-ORF73 MHV-68 as in C, then stained for the ORF65 capsid component with mAb MG-12B8. The data are from 1 of 2 equivalent experiments.

Infected BAC-eGFP^−^ macrophages can be revealed by LPS treatment, which activates the HCMV IE1 promoter [38]. With an infection multiplicity of 3PFU/cell, LPS induced BAC-eGFP expression in almost all MHV-68-exposed, MHC class II^lo^ DCs (Fig. 3A). Only MHC class II^hi^ DCs remained eGFP^−^, suggesting that these were not infected. These DCs also remained gM-eGFP^− ^(Fig. 3A). Unlike BAC-eGFP^+^ DCs, gM-eGFP^+^ DCs largely disappeared within 6 h of LPS treatment. The activation of HCMV IE1 transcription by LPS therefore did not indicate a shift to MHV-68 lytic infection - if anything, the opposite, as lytic infection seemed incompatible with LPS stimulation. Immunofluorescence confirmed that LPS treatment led to a complete loss of lytically infected DCs (Fig. 3B). The only gM-eGFP fluorescence left was limited and localized, the pattern of endocytic uptake rather than new gene expression (Fig. 2B). The significance of the LPS-triggered death of lytically infected DCs is unclear - these stimuli are hardly a physiological combination - but it made clear that the functional effect of such stimuli on virus-exposed DCs cannot be interpreted without some measure of viral gene expression. It appeared that an infection multiplicity of 3PFU/cell infected all immature DCs, the majority latently, while very few mature DCs were infected.

**Figure 4:**
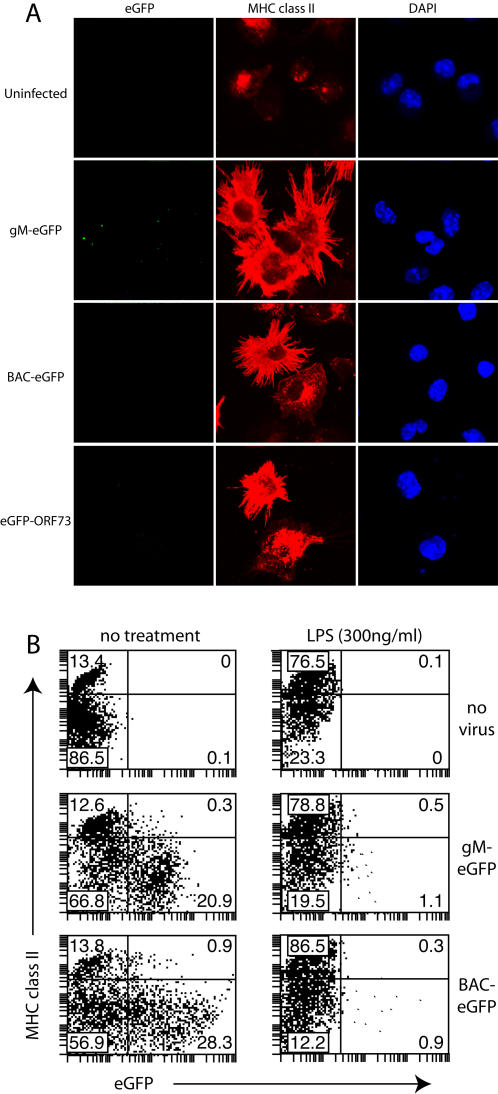
No sign of MHV-68 infection in mature DCs. A. DCs were left uninfected or exposed to gM-eGFP, BAC-eGFP or eGFP-ORF73 MHV-68 (3PFU/cell, 22 h). The non-adherent cells were centrifuged onto cover slips, then stained for MHC class II expression and also examined for eGFP expression/uptake. The data are from 1 of 2 equivalent experiments. B. Bone marrow-derived DCs were incubated or not with LPS to trigger maturation, then exposed to BAC-eGFP^+^ or gM-eGFP^+^ MHV-68 as shown (3PFU/cell). 22 h later, CD11c^+^ cells were analyzed for surface MHC class II expession and virus-driven eGFP.

As a further measure of latent infection we generated MHV-68 with eGFP-tagged ORF73 - its episome maintenance protein [42], [43]. ORF73 is not necessarily transcribed in every latently infected cell - transcription of the functionally homologous EBNA-1 is linked to cell division [44] - but it is currently the best available marker. EGFP fluorescence was faint (Fig. 3C), but almost all (>90%) the DCs exposed to eGFP-ORF73 MHV-68 (3PFU/cell) showed punctate nuclear fluorescence, consistent with the distribution of the homologous KSHV ORF73 [45]. All DCs with nuclear ORF65 staining (lytic infection) were also eGFP-ORF73^+^, but most eGFP-ORF73^+^ DCs were ORF65^−^. The eGFP-ORF73^+^ORF65^−^ DCs were presumably latently infected. Again, with an infection multiplicity of 3PFU/cell, the only eGFP-ORF73^−^ cells were those with high MHC class II expression (Fig. 4A). Interestingly, the lytically and latently infected MHC class II^lo^ cells all became adherent. Thus, in uninfected cultures both mature and immature DCs were largely non-adherent, with 90% of the non-adherent DCs being immature; but in infected cultures, only the mature (uninfected) DCs remained non-adherent (Fig. 4A). This did not reflect an increase in maturation, but rather a selective adherence to plastic of the immature, infected DCs.

We addressed the infectibility of mature DCs further by exposing immature DCs to LPS or not 5 h before exposing them to MHV-68 (Fig. 4B). In contrast to the immature DCs, the LPS-matured DCs failed to express either BAC-eGFP or gM-eGFP. Thus both spontaneously matured and deliberately matured DCs resisted MHV-68 infection.

### Lytically infected DCs down-regulate MHC class I-restricted antigen presentation

**Figure 5:**
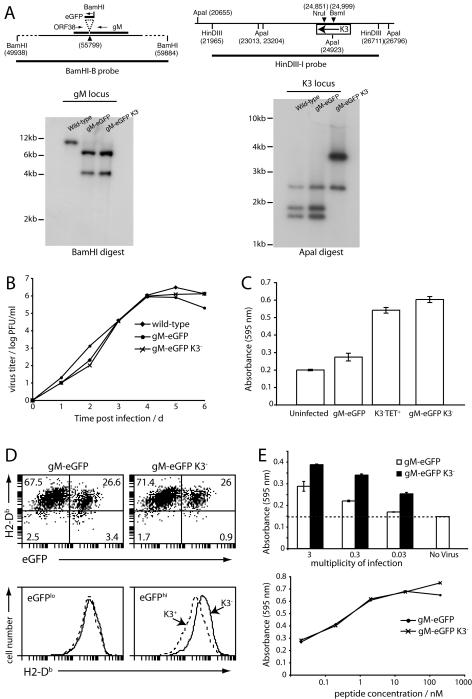
Characterization of K3 function in MHV-68-infected DCs. A. K3 was disrupted on the gM-eGFP background by introducing a deletion after its first transmembrane domain. Wild-type, K3^+^gM-eGFP and K3^−^gM-eGFP viruses were analyzed by Southern blotting. The predicted alterations to the gM and K3 loci are shown. Thus, gM-eGFP insertion donwstream of gM introduces a BamHI restriction site such that the 10 kb genomic BamHI fragment is cut into 4.1 kb and 6.6 kb fragments. The K3 deletion removes an ApaI site, such that 1.7 kb and 1.9 kb genomic fragments are combined into a single 3.4 kb fragment. B. BHK-21 cells were infected (0.01PFU/cell) as indicated. Replicate cultures were frozen at each time point. All were then assayed for virus titer by plaque assay. C. H2^b^ MEF-1 fibroblasts were left uninfected or infected (2PFU/cell, 4 h) with K3^+^ or K3^−^ gM-eGFP viruses or a gM-eGFP^−^ K3 mutant (K3^−^TET^+^) [20] as a control. The cells were then washed and incubated overnight with the 49100.2 T cell hybridoma, which recognizes an H2-D^b^-restricted MHV-68 lytic epitope and produces β-galactosidase upon activation. The cells were lysed in 1% NP-40 and beta-galactosidase activity measured with CPRG. Each bar shows mean ± SD absorbance readings from triplicate cultures. The data are from 1 of 3 equivalent experiments. D. DCs were infected (3PFU/cell, 22 h) with K3^+^ or K3^−^ gM-eGFP MHV-68 as indicated, then and stained for cell surface H2-K^b^ expression and eGFP expression by flow cytometry. Each graph shows gated CD11c^+^ cells. In the dot plots, the number shows the percentage of all CD11c^+^ cells in each quadrant. The histograms show equivalent data, but gated according to high or low gM-eGFP expression. The data are from 1 of 3 equivalent experiments. E. H2^b^ DCs were infected with K3^+^ or K3^−^ viruses as indicated for 4 h, washed and incubated overnight with the MHV-68 p56-specific 49100.2 T cell hybridoma. β-galactosidase expression was then measured with CPRG. Mean ± DC absorbance values of triplicate cultures are shown. Duplicate samples were coated with reducing concentrations of p56 peptide and assayed as above. The data are from 1 of 3 equivalent experiments.

A key aspect of the MHV-68-infected DC phenotype is MHC class I-restricted antigen presentation. The MHV-68 M3 can inhibit CD8^+^ T cell migration [46], but only K3 has been shown to inhibit CD8^+^ T cell recognition [7]. In order to compare K3^+^ and K3^−^ gM-eGFP^+^ viruses, we truncated K3 after its first transmembrane domain in the gM-eGFP BAC (Fig. 5A). This inactivates it completely [8]. Southern blots confirmed the predicted loss of an ApaI site in the deleted K3 segment, as well as the diagnostic BamHI site between gM and its C-terminal eGFP tag (Fig. 5A). The growth of K3^+^ and K3^−^ gM-eGFP mutants was indistinguishable from that of wild-type MHV-68 (Fig. 5B). As expected, K3 disruption increased lytic antigen presentation by fibroblasts infected with gM-eGFP MHV-68, much like a K3 knockout on the wild-type BAC background (Fig. 5C).

Lytically infected (gM-eGFP^hi^) DCs down-regulated MHC class I expression relative to uninfected or latently infected (gM-eGFP^lo^) DCs (Fig. 5D). This down-regulation was K3-dependent, as it was not seen with the gM-eGFP K3^−^ mutant. DCs infected with the K3 mutant also showed better lytic antigen presentation than those infected with wild-type (Fig. 5E). Exogenous peptide was presented much the same, consistent with latently infected DCs not expressing K3. A comparison of the infected cells and peptide-pulsed cells in Fig. 5E indicated that K3 reduced endogenous p56 presentation approximately 10-fold. The non-zero antigen presentation of K3^+^ MHV-68 in Fig. 5E was unsurprising. Not only is K3 unlikely to be 100% efficient, but non-lytic DCs can presumably still cross-present the virion and infected cell debris. This result emphasizes that K3 is unlikely to have global effect on immune priming in MHV-68 infection. It acts mainly to disguise lytically infected DCs.

### K3 protects lytically infected DCs against CD8^+^ T cell-mediated lysis

**Figure 6:**
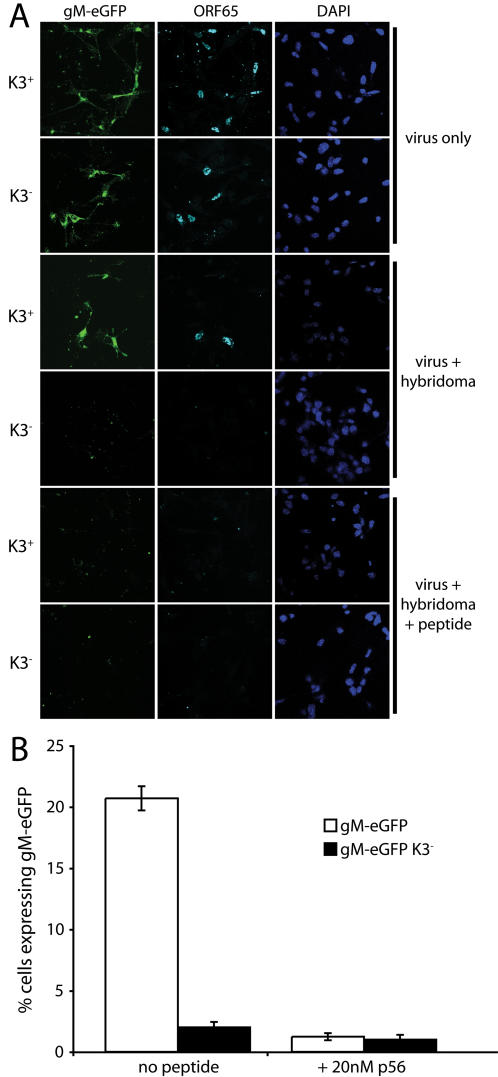
K3-dependent CD8^+^ T cell evasion by MHV-68-infected DCs. A. H2^b^ DCs were infected (3PFU/cell, 16 h) with K3^+^ or K3^−^ gM-eGFP MHV-68 viruses and then co-cultured with the H2-D^b^-restricted, p56-specific 49100.2 hybridoma cells with or without 20 nM p56 peptide for a further 6 h. The DCs were then washed, fixed and stained for ORF65 capsid expression with mAb MG-12B8. The hybridoma cells are partially adherent, so where hybridoma cells were added they may contribute to the DAPI staining. B. Mean ± SD values of gM-eGFP^+^ cell counts in randomly selected fields (>20 each). Each number is expressed relative to the number for infection without hybridoma cells. Thus, “20%” means that the treatment arm had 20% the number of eGFP^+^ cells per field seen when hybridoma cells were not added. The data are from 1 of 3 equivalent experiments.

To test further the impact of K3 on CD8^+^ T cell recognition of lytically infected DCs, we made use of the fact that the MHV-68 p56 epitope-specific hybridoma 49100.2 retains cytotoxic effector function. We infected DCs overnight with K3^−^ or K3^+^ gM-eGFP viruses, added hybridoma cells with or without 20 nM p56 peptide for a further 7 h, then counted the ORF65^+^gM-eGFP^+^ cells remaining (Fig. 6). K3 expression substantially protected lytically infected cells against recognition by 49100.2 T cells unless exogenous peptide was also added.

**Figure 7:**
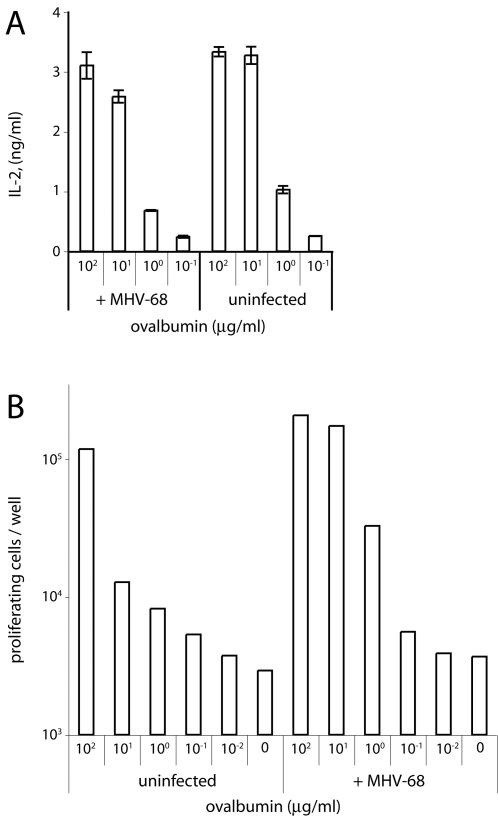
Antigen presentation by mixed infected DC cultures. A. H2^d^ DCs were infected with MHV-68 (3PFU/cell, 18 h). Ovalbumin was then added to the DCs for 2 h and removed. DO.11.10 hybridoma cells (CD4^+^, ovalbumin-specific, IA^d^-restricted) were then added for a further 15 h. IL-2 in cell supernatants was then measured by ELISA. Each bar shows mean ± SD values of triplicate cultures. The data are from 1 of 3 equivalent experiments. B. H2^b^ DCs were infected with MHV-68 (3PFU/cell, 24 h). Ovalbumin was then added to the DCs for 6 h and removed. CFSE-labelled OT-I transgenic T cells from lymph nodes (CD8^+^, ovalbumin-specific, H2-K^b^-restricted) were then added for a further 3 d. The fraction of proliferating CD8^+^ cells (based on loss of CFSE staining) was then determined by flow cytometry. The data are from 1 of 3 equivalent experiments.

In contrast to the antigen presenting defect of lytically infected DCs, unfractionated MHV-68-infected DC cultures processed and presented exogenous ovalbumin fairly normally to both I-E^d^-restricted DO.11.10 T cells and H2-K^b^-restricted OT-I T cells (Fig. 7). This illustrates how mixed culture results can be misleading. It was clear that without establishing reasonably uniform viral gene expression in the population under study, only very limited conclusions can be drawn about viral gene functions.

### MHV-68 down-regulates CD86 expression on lytically infected DCs

**Figure 8:**
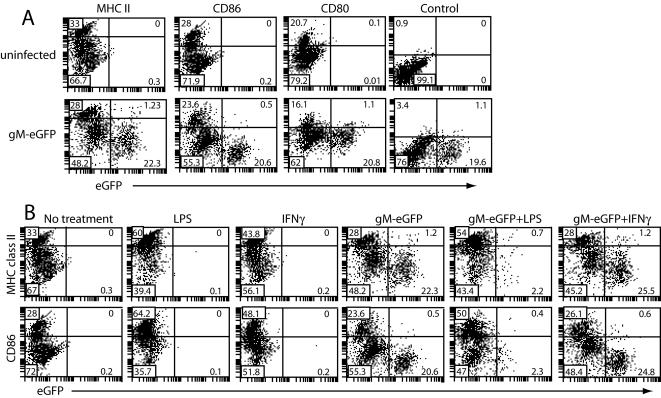
CD86 down-regulation by lytically infected DCs. A. DCs were left uninfected or infected (3PFU/cell, 22 h) with gM-eGFP^+^ MHV-68 and then assayed for cell surface CD11c, MHC II, CD86 and CD80 expression by flow cytometry. Control = isotype-matched irrelevant antibody. Plots show CD11c^+^ cells and are representative of 4 independent experiments. B. DCs were left uninfected or infected (3PFU/cell, 14 h), then treated with LPS (250 ng/mL), IFNγ (200 U/mL) or nothing for a further 7 h. The cells were then stained for surface CD11c, CD86 and MHC class II. Plots show CD11c^+^ cells and are representative of 3 independent experiments. CD11c positive cells are shown and are representative of 3 independent experiments.

We also looked for lytic cycle down-regulation of other DC molecules involved in antigen presentation (Fig. 8A). CD80 was unaffected by MHV-68 infection. However, CD86 expression was noticeably less on gM-eGFP^hi^ cells than on uninfected or gM-eGFP^lo^. Even ignoring the DCs with high CD86 expression, which resist MHV-68 infection (Fig. 4), CD86 expression was clearly less on the gM-eGFP^hi^ cells compared to gM-eGFP^−^ and gM-eGFP^lo^. gM-eGFP^hi^ cells also down-regulated MHC class II expression somewhat relative to gM-eGFP^lo^ cells. Neither effect was due to K3 (data not shown).

We tested further whether LPS or IFN-γ might influence CD86 or MHC class II expression on lytically infected DCs (Fig. 8B). IFN-γ upregulated CD86 on uninfected but not gM-eGFP^hi^ (lytically infected) DCs. gM-eGFP^lo^ DCs responded weakly. These DCs are probably latently infected: they correspond to those in Fig. 2B that have endocytosed virions but not yet initiated lytic infection, and to the DCs in Fig. 3C that are eGFP-ORF73^+^ORF65^−^. LPS upregulated MHC class II and CD86 expression on both uninfected and gM-eGFP^lo^ cells. Its main effect on gM-eGFP^hi^ cells was, as in Fig. 3, to trigger their destruction. Thus, latently infected DCs responded fairly normally to LPS but were impaired in their response to IFN-γ. Lytically infected DCs responded abnormally to both: IFN-γ had no effect and LPS triggered cell death.

## Discussion

Herpesvirus latency and lytic replication are very different states, so the first step in defining infected DC phenotypes must be to distinguish them, both from uninfected cells and from each-other. MHV-68 established a predominantly latent infection in immature, bone marrow-derived DCs, much as it does in peritoneal macrophages [38]. Latency was not stably maintained, and viral reactivation killed most of the DCs in infected cultures over a few days. Lytically infected DCs showed a K3-dependent inhibition of MHC class I-restricted antigen presentation, a K3-independent down-regulation of CD86 expression, and a highly abnormal response to activation signals. Conclusions about latency must be more guarded. We found that latently infected DCs respond poorly to IFN-γ but fairly normally to LPS, at least over a 6 h time frame. Thus, it was clear that latent infection disrupts DC responses much less than lytic infection does, but MHV-68 latency may encompass more than one viral gene expression program. Understanding the latently infected DC phenotype requires a better definition of viral gene expression.

Hochreiter et al. found that MHV-68 inhibits DC responses to maturation signals [47], but they did not distinguish lytic from latent infection, so whether they observed mainly the lytic or the latent infection phenotype is unclear. Specifically, our data contradict their assumption that transcription from an HCMV IE1 promoter at the left end of the MHV-68 genome corresponds to lytic infection. Instead, this promoter was regulated independently of endogenous viral gene expression. This has important implications for studies that use HCMV IE1 promoters to drive MHV-68 gene expression in vivo. MHV-68 specifically protects its episome maintenance protein from MHC class I-restricted antigen presentation [18]. An HCMV IE1 promoter would give gene expression independent of such evasion and could therefore make latently infected cells vulnerable to immune elimination, independent of the function of the gene being expressed.

Bone marrow-derived DC cultures exposed to MHV-68 contained not only lytic and latent infections, but also uninfected cells. Specifically, mature DCs (MHC class II^hi^CD86^hi^) showed neither gM-eGFP expression (indicative of lytic infection), eGFP-ORF73 expression (lytic or latent infection) nor LPS-inducible BAC-eGFP expression (independent of MHV-68 gene expression). Thus, while immature DCs were either lytically or latently infected, mature DCs were largely uninfected, perhaps because they drastically reduce endocytosis [48], the route by which MHV-68 normally infects [49]. The presence of mature, uninfected DCs in infected cultures obviously makes assays that do not distinguish between latent infection and no infection very hard to interpret.

Our finding that K3 disruption markedly enhances lytic epitope presentation contradicts the speculation of Flano et al. that K3 does not work in DCs. It seems likely that Flano et al. and Hochreiter et al. both failed to notice K3 function in DCs because they did not distinguish lytic from latent infection. The lytic antigen presentation observed by Flano et al. emphasizes further the problem of using in vitro assays as absolute measures, rather than to compare wild-type and knockout viruses (or DCs). Immune evasion is rarely absolute, and cell debris and defective virus particles provide abundant antigen for cross-presentation. Some T cell response is therefore unsurprising. Without a suitable comparison, its significance is easily over-interpreted.

How do the MHV-68-infected DC phenotypes fit with what we know of pathogenesis? It is unlikely that DC infection has much global effect on antigen presentation. Relatively few DCs are infected [23], so cross-priming should still operate. Indeed, the magnitude of acute, MHV-68-specific CD8^+^ T cell responses is quite in keeping with that made to other viral infections [24]. K3-deficient MHV-68 stimulates stronger CD8^+^ T cell responses than wild-type [20], but this is as likely to reflect more stimulation of primed cells as more priming. Herpesviruses seem to rely mainly on effector cell evasion. Thus, rather than limiting immune priming K3 may help lytically infected DCs to evade CD8^+^ T cell recognition once priming has occurred. What might lytic DC infection contribute to pathogenesis that it needs to be protected? One possibility is virus movement. DCs probably get infected in peripheral sites, where viral lytic replication is abundant [26]. DC migration to draining lymph nodes followed by viral reactivation would then be one way to infect B cells [50]. Lytically infected DCs may also secrete the M3 chemokine binding protein to provide bystander protection for latently infected B cells [51]. In both settings, a lack of K3 would impair latency establishment by predisposing lytically infected DCs to immune elimination. This could in part explain the latency amplification deficit of K3-deficient mutants. Even when an in vivo phenotype and a biochemical function are known, it would seem that linking the two is not necessarily straightforward.
